# MicroRNA-26b suppresses autophagy in breast cancer cells by targeting DRAM1 mRNA, and is downregulated by irradiation

**DOI:** 10.3892/ol.2021.12460

**Published:** 2021-01-12

**Authors:** Cuida Meng, Yang Liu, Yannan Shen, Shuchun Liu, Zhicheng Wang, Qingsheng Ye, Hongyang Liu, Xiaodong Liu, Lili Jia

Oncol Lett 15: 1435-1440, 2018; DOI: 10.3892/ol.2017.7452

Subsequently to the publication of this paper, an interested reader drew to the authors’ attention that a pair of data panels featured in Fig. 2B (specifically, those showing the data from the ‘mimic miR-26b + 4 Gy’ and ‘inhibitor miT-26b + 0 Gy’ experiments) appeared to be strikingly similar.

The authors have re-examined their data, and realized that Fig. 2B was assembled incorrectly (the panel showing the data for the ‘mimic miR-26b + 4 Gy’ experiment had been accidentally duplicated in the figure). The revised version of Fig. 2, containing the correct data for the ‘inhibitor miT-26b + 0 Gy’ experiment in Fig. 2B, is shown opposite. The authors regret the error that was made in the preparation of the published figure, and confirm that this error did not seriously affect the conclusions reported in the paper. The authors are grateful to the editor of *Oncology Letters* for allowing them the opportunity to publish a Corrigendum, and all the authors agree to this Corrigendum. Furthermore, they apologize to the readership for any inconvenience caused.

## Figures and Tables

**Figure 2.**
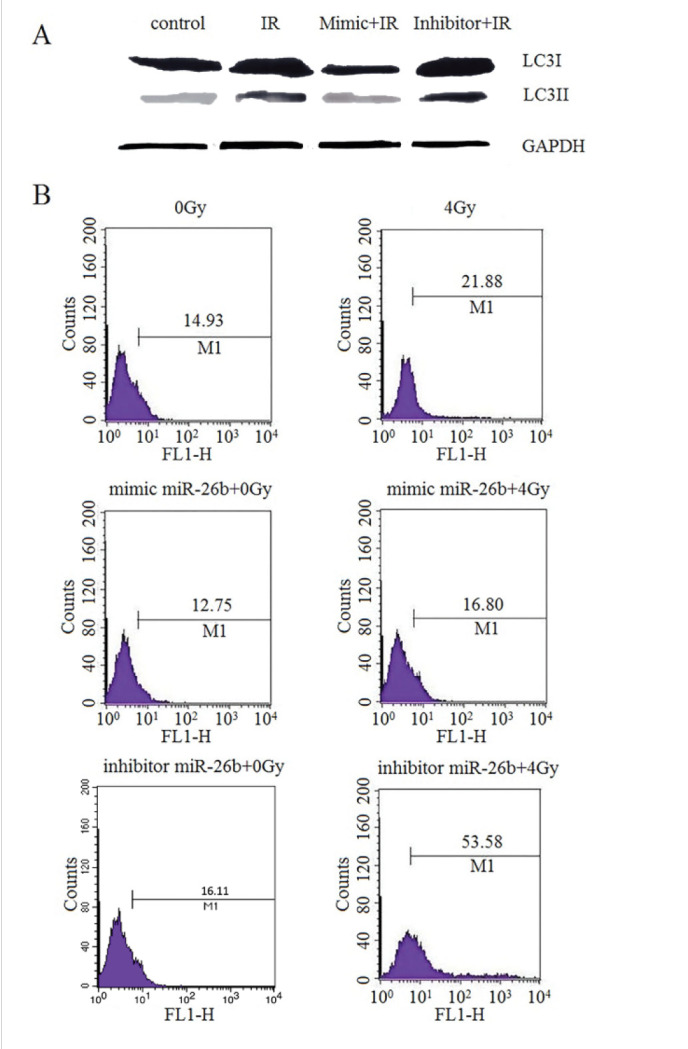
miR-26b regulated autophagy in MCF7 cells exposed to IR. MCF7 cells that were transfected with NC, miR-26b mimic or the miR-26 inhibitor were subsequently exposed to IR. (A) miR-26b promoted the LC3II protein expression induced by IR. (B) MCF7 cells were exposed to 4 Gy of IR. Staining with monodansylcadaverine was detected by flow cytometry at 24 h. miR, microRNA; IR, ionizing radiation; IR, ionizing radiation; NC, negative control; LC3II, light chain 3-II.

